# Outcomes From Opportunistic Salpingectomy for Ovarian Cancer Prevention

**DOI:** 10.1001/jamanetworkopen.2021.47343

**Published:** 2022-02-09

**Authors:** Gillian E. Hanley, Celeste Leigh Pearce, Aline Talhouk, Janice S. Kwon, Sarah J. Finlayson, Jessica N. McAlpine, David G. Huntsman, Dianne Miller

**Affiliations:** 1 Division of Gynaecologic Oncology, Department of Gynaecology and Obstetrics, The University of British Columbia, Vancouver, British Columbia, Canada; 2 Vancouver General Hospital Research Pavilion, Vancouver, British Columbia, Canada; 3 Department of Epidemiology, University of Michigan School of Public Health, Ann Arbor; 4 Pathology & Laboratory Medicine, The University of British Columbia, Vancouver, British Columbia, Canada

## Abstract

**Importance:**

Opportunistic salpingectomy (OS), which is the removal of fallopian tubes during hysterectomy or instead of tubal ligation without removal of ovaries, is recommended to prevent ovarian cancer, particularly serous ovarian cancer. However, the effectiveness of OS is still undetermined.

**Objective:**

To examine observed vs expected rates of ovarian cancer among individuals who have undergone OS.

**Design, Setting, and Participants:**

This is a population-based, retrospective cohort study of all individuals in British Columbia, Canada, who underwent OS or a control surgery (hysterectomy alone or tubal ligation) between 2008 and 2017, with follow-up until December 31, 2017. Those with any gynecological cancer diagnosed before or within 6 months of their procedure were excluded. Data analysis was performed from April to August 2021.

**Exposures:**

Removal of both fallopian tubes at the time of hysterectomy or instead of tubal ligation while leaving ovaries intact.

**Main Outcomes and Measures:**

An ovarian cancer diagnosis listed in the British Columbia Cancer Registry. Age-specific rates of epithelial and serous ovarian cancer in the control group were combined with the specific follow-up time in the OS group to calculate expected numbers (and 95% CIs) of ovarian cancers in the OS group. These were compared with observed numbers. Age-adjusted expected and observed numbers of breast and colorectal cancers were also examined in the OS group.

**Results:**

There were 25 889 individuals who underwent OS (mean [SD] age, 40.2 [7.1] years; median [IQR] follow-up, 3.2 [1.6-5.1] years) and 32 080 who underwent hysterectomy alone or tubal ligation (mean [SD] age, 38.2 [7.9] years; median [IQR] follow-up, 7.3 [4.6-8.7] years). There were no serous ovarian cancers in the OS group and 5 or fewer epithelial ovarian cancers. The age-adjusted expected number was 5.27 (95% CI, 1.78-19.29) serous cancers and 8.68 (95% CI, 3.36-26.58) epithelial ovarian cancers. Age-adjusted expected vs observed numbers of breast cancers (22.1 expected vs 23 observed) and colorectal cancers (9.35 expected vs 8 observed) were not significantly different.

**Conclusions and Relevance:**

In this cohort study, the OS group had significantly fewer serous and epithelial ovarian cancers than were expected according to the rate at which they arose in the control group. These findings suggest that OS is associated with reduced ovarian cancer risk.

## Key Points

**Question:**

Is opportunistic salpingectomy associated with fewer than expected ovarian cancers?

**Findings:**

This population-based cohort study included 25 889 individuals who underwent opportunistic salpingectomy and 32 080 individuals who underwent hysterectomy alone or tubal ligation. There were no serous ovarian cancers among individuals in the opportunistic salpingectomy group, which was significantly lower than the age-adjusted expected rate of 5.27 serous cancers.

**Meaning:**

The opportunistic salpingectomy group had significantly fewer serous ovarian cancers than expected, suggesting that opportunistic salpingectomy is associated with reduced ovarian cancer risk.

## Teaser

This cohort study examines observed vs expected rates of ovarian cancer among individuals who have undergone opportunistic salpingectomy.

## Introduction

Approximately 70% of sporadic and nearly all ovarian cancers in *BRCA* variant carriers are high-grade serous carcinomas (HGSCs),^1^ which is the most lethal of the 5 main histotypes and has a 5-year survival rate less than 50%.^2^ Although the general population lifetime risk of ovarian cancer is 1.4%,^3^ individuals with an inherited germline *BRCA1* or *BRCA2* variant have average cumulative risks of 40% to 75% and 8% to 34%, respectively.^4^ In *BRCA 1/2* variant carriers, bilateral salpingo-oophorectomy is recommended, which reduces the risk of ovarian or fallopian tube cancers by 80%.^5^ Removal of the ovaries is not recommended for the general population because it is associated with increased total mortality, coronary heart disease, and osteoporosis.^6^ Thus, a different preventive strategy is needed for individuals at average risk, who account for 80% of cases of HGSCs.

The recent understanding that HGSC often originates in the fallopian tube^7,8^ has led to a primary prevention opportunity for the general population—namely, opportunistic salpingectomy (OS). OS collectively refers to the removal of the fallopian tubes at the time of hysterectomy or instead of tubal ligation, while leaving the ovaries intact. In 2010, the British Columbia (BC) ovarian cancer research team launched a province-wide strategy asking gynecologists to discuss OS with their patients as an ovarian cancer prevention strategy. The same recommendation has since been made in many countries, including Canada, the US, and the UK for individuals without identified genetic factors associated with increased risk of ovarian cancer.^9,10,11,12^ Research has shown that OS is safe, both in terms of perioperative adverse events^13^ and minor complications,^14^ there are no indications of an earlier age of onset of menopause following OS,^15^ and it is cost-effective.^16^

Some retrospective data from individuals who underwent bilateral salpingectomy for conditions such as hydrosalpinx and pelvic inflammatory disease have suggested decreased risk of ovarian cancer among individuals without fallopian tubes.^17,18,19^ However, we hypothesize that OS is associated with more protection than salpingectomies done for diseases that directly affect and distort the fallopian tube, because the intent of OS is complete removal of the fimbriated end of the fallopian tube, which may not occur when salpingectomy is done for other indications. Finally, these historical studies did not use the appropriate control groups, as OS is recommended only for individuals already undergoing gynecological surgery, and both hysterectomy and tubal ligation are associated with protection against ovarian cancer.^20^ Here, we examine observed rates of ovarian cancer and compare these with expected rates (based on age-adjusted rates of ovarian cancer in the control group of individuals who underwent hysterectomy alone or tubal ligation).

## Methods

This population-based, retrospective, cohort study examined data on all residents of the Canadian province of BC (population, 5 million). All individuals who underwent a hysterectomy or tubal sterilization in BC between 2008 and 2017 were included. Approvals were obtained from all relevant data stewards, and access to the Consolidation file, the BC Cancer Registry, the Discharge Abstract Database, and the BC Cancer Agency Screening Program was facilitated through Population Data BC. More details, including citations to data sources, are presented in the eTable in the Supplement. Ethics approval was obtained from the University of British Columbia’s Behavioral Research Ethics Board. Approval by the ethics board and the BC data stewards for use of deidentified administrative data files includes a waiver of informed consent from participants. This study follows the Strengthening the Reporting of Observational Studies in Epidemiology (STROBE) reporting guideline.^21^

### Exposure

Individuals who underwent any of the relevant surgical procedures were identified using the Canadian Classification of Health Intervention codes. This system separately identifies each procedure performed during the same surgery, and a person undergoing a hysterectomy with bilateral salpingectomy has both a code indicating the removal of the uterus and one indicating the removal of the fallopian tubes. Individuals with a diagnosis of any gynecological cancer before or within 6 months of their surgery were excluded, as this cancer was likely present at the time of surgery. Individuals were stratified into 2 groups according to their procedures: (1) those who underwent OS, meaning they had a hysterectomy with a salpingectomy but no oophorectomy, or they had a bilateral salpingectomy alone with a diagnosis code indicating the procedure was for sterilization (*International Statistical Classification of Diseases, Tenth Revision, Clinical Modification [ICD-10-CM] *code Z.30.2), and (2) those who underwent control surgical procedures, which included those who had undergone a hysterectomy with no concomitant oophorectomy or salpingectomy and anyone who underwent a tubal ligation.

### Outcome

In BC, cancer is a reportable disease, and all cases are entered into the provincial cancer registry. The registry sources include hematology and pathology reports, death certificates, hospital reports, and cancer treatments (see the eTable in the Supplement for more details). All ovarian cancers were identified using the *International Classification of Diseases for Oncology (ICD-O) *codes for ovarian cancer (*ICD-O* code C56.X), fallopian tube cancer (*ICD-O* code C57.0), or peritoneal cancer, not otherwise specified (*ICD-O* code C48.2). All epithelial ovarian cancers diagnosed after a surgery of interest were included, and borderline tumors were excluded. *ICD-O* morphology codes were used to identify the histotype of the ovarian cancers according to the algorithm published by Peres et al.^22^ The grade data were incomplete, and all serous cancers were presented together rather than as low grade and high grade; however, 95% of serous cancers are HGSCs. To examine whether differences in the observed and expected ovarian cancers in the OS group might be explained by underlying differences in the likelihood of getting cancer associated with health status, lifestyle, genetics, and so forth, differences in observed and age-adjusted expected numbers of breast cancer (*ICD-O* code C50) and colorectal cancer (*ICD-O* code C18.X) but excluding cancer of the appendix (*ICD-O* code C18.1) were also examined.

### Potential Confounders

We examined how the groups differed with respect to potentially important confounders, including age at the time of surgery, income quintiles, parity, gravidity, history of oral contraceptive pill use and total mean days of oral contraceptive use, presence of a known *BRCA* variant, and the presence of benign gynecological conditions at the time of surgery, including endometriosis (*ICD-10-CM* code N80.X), leiomyoma (*ICD-10-CM* code D25.X), benign ovarian or uterine neoplasm (*ICD-10-CM* codes D26.X, D27.X, and D28.7), abnormal bleeding (*ICD-10-CM* codes N92.X and N93.X), pelvic organ prolapse (*ICD-10-CM* code N81.X), pelvic inflammatory disease (*ICD-10-CM* codes N73.X and N74.X), and hydrosalpinx (*ICD-10-CM* code N70.X).

### Statistical Analysis

For privacy reasons, data steward agreements require that we not publish cell sizes between 1 and 5. The number of observed epithelial and serous ovarian cancers in the OS group are presented according to privacy requirements. We also present the number of observed breast and colorectal cancers. These observed numbers were then compared with the number expected on the basis of age-adjusted (in 5-year age groups) rates in the control group multiplied by the person-time contribution in the OS group. Given the low number of ovarian cancers in both groups, statistical models were not run. Instead the distribution of the potential confounders across the OS and control groups and their standardized differences were presented. A difference between covariates was considered meaningful if the standardized difference was greater than 0.1.^23^

The age-adjusted rates of serous ovarian cancers were also used to project the number of expected serous ovarian cancers in the OS group 5 and 10 years beyond our study period if serous ovarian cancers were to occur at the same rate as in the control group. To project, we used data from the cohort included in our control group and adjusted the person-time contribution in each age group as individuals in the cohort age. Data were analyzed using Stata statistical software version 16 (StataCorp). Data analysis was performed from April to August 2021.

## Results

**Figure 1.**
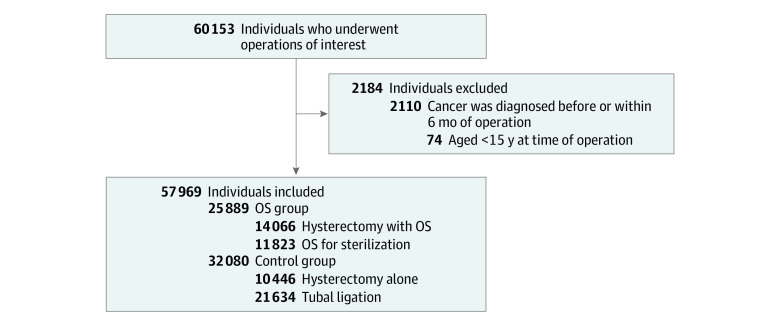
Flow Diagram of Study Cohort This study examined ovarian cancers in individuals who underwent opportunistic salpingectomy (OS) compared with a control group who underwent hysterectomy alone and tubal ligation.

Before exclusions, there were 60 153 individuals who underwent any of the surgical procedures of interest. After exclusion of 74 individuals who were younger than age 15 years at the time of surgery and 2110 individuals with cancers that were diagnosed before or within 6 months of surgery, there were 25 889 individuals (mean [SD] age, 40.2 [7.1] years) in the OS group, including 14 066 who underwent hysterectomy with OS and 11 823 who underwent OS for sterilization. There were 32 080 individuals (mean [SD] age, 38.2 [7.9] years) in the control group, including 10 446 individuals who underwent hysterectomy alone and 21 634 who underwent tubal ligation (Figure 1). The Table compares the characteristics of the OS groups (hysterectomy with OS and OS for sterilization) with the control groups (hysterectomy alone and tubal ligation). There were no meaningful differences in any oral contraceptive pill use (18 098 individuals [69.9%] in the OS group vs 21 414 individuals [66.8%] in the control group), duration of oral contraceptive pill use (OS group, mean [SD], 757 [1091] days and median [IQR], 265 [0-1080] days; control group, mean [SD], 662 [965] days and median [IQR], 218 [0-960] days), and *BRCA* variant rates between the groups (27 individuals [0.10%] in the OS group vs 34 individuals [0.11%] in the control group). Individuals who underwent OS were older at the time of surgery (mean [SD], 40.2 [7.1] vs 38.2 [7.9] years), had fewer live births (mean [SD], 1.74 [1.29] vs 2.03 [1.33] live births) and fewer pregnancies (mean [SD], 2.26 [1.87] vs 2.63 [1.93] pregnancies), and were more likely to have endometriosis (3301 individuals [12.8%] vs 2279 individuals [7.1%]) than those in the control group. There was longer follow-up in the control group vs the OS group (median [IQR], 7.3 [4.6-8.7] vs 3.2 [1.6-5.1] years).

**Table.** Characteristics of the Opportunistic Salpingectomy Group and Those Who Underwent Hysterectomy Alone or Tubal Ligation

Characteristic	Patients, No. (%)		Standardized mean difference
	Hysterectomy alone or tubal ligation (n = 32 080)	Opportunistic salpingectomy (n = 25 889)	
Age at time of surgery, mean (SD), y	38.2 (7.9)	40.2 (7.1)	0.2743
Duration of follow-up, median (IQR), y	7.3 (4.6-8.7)	3.2 (1.6-5.1)	0.8987
Income quintile			
Patients, No.	27 488	13 592	
1	5869 (21.4)	2718 (20.0)	0.0675
2	6185 (22.5)	2782 (21.2)	
3	5769 (21.0)	2887 (21.2)	
4	5327 (19.4)	2867 (21.1)	
5	4338 (15.8)	2338 (17.2)	
Parity, live births, mean (SD), No.	2.03 (1.33)	1.74 (1.29)	0.2239
Pregnancies, mean (SD), No.	2.63 (1.93)	2.26 (1.87)	0.1965
Oral contraceptive pill use	21 414 (66.8)	18 098 (69.9)	0.0678
Duration of oral contraceptive pill use, d			
Mean (SD)	662 (965)	757 (1091)	0.0923
Median (IQR)	218 (0-960)	264 (0-1080)	
*BRCA* variant	34 (0.11)	27 (0.10)	0.0023
Endometriosis	2279 (7.1)	3301 (12.8)	0.1897
Uterine leiomyoma	3760 (11.7)	6474 (25.0)	0.3483
Benign uterine or ovarian neoplasm	225 (0.7)	355 (1.4)	0.0661
Prolapse	2127 (6.6)	1314 (5.1)	0.0663
Abnormal bleeding	6919 (21.6)	9395 (36.3)	0.3290
Pelvic inflammatory disease	606 (1.9)	746 (2.9)	0.0628
Hydrosalpinx	122 (0.4)	209 (0.8)	0.0556
Polycystic ovary syndrome	43 (0.1)	50 (0.19)	0.0146

**Figure 2.**
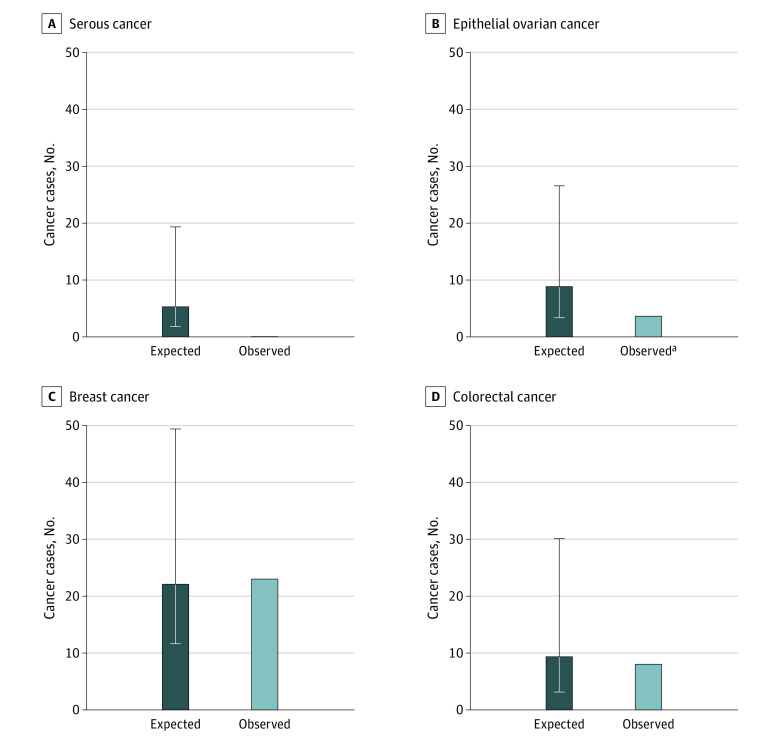
Numbers of Expected vs Observed Cancers in the Opportunistic Salpingectomy Group Error bars denote 95% CIs. ^a^Denotes a cell size of less than or equal to 5, not an exact number.

Figure 2A illustrates that there were no serous cancers in the OS group by the end of follow-up. Given the age-adjusted rate at which serous ovarian cancers occurred in the control group and the follow-up time in the OS group, 5.27 (95% CI, 1.78-19.29) serous cancers were expected. The same is true for all epithelial ovarian cancers. The expected number of epithelial ovarian cancers in the OS group was 8.68 (95% CI, 3.36-26.58) cancers, and the actual number was less than or equal to 5 (exact number not presented to protect patient privacy) (Figure 2B). In contrast, there were 15 serous cancers in the control group, and 21 epithelial ovarian cancers (including 6 nonserous cancers). Figures 2C and 2D show no significant differences between expected and observed numbers of breast or colorectal cancers in the OS group. The age-adjusted expected number of breast cancers in the OS group was 22.1 (95% CI, 11.62-49.37) cancers, and 23 breast cancers were observed in this group. The age-adjusted expected number of colorectal cancers in the OS group was 9.35 (95% CI, 3.13-30.11) cancers, and 8 cancers were observed.

**Figure 3.**
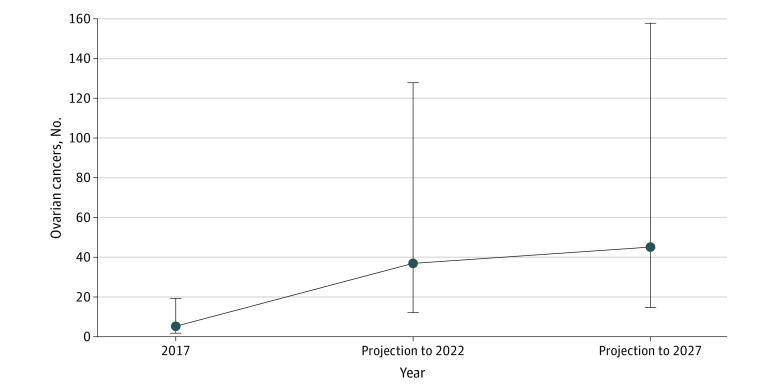
Projected Expected Numbers of Serous Ovarian Cancers in the Opportunistic Salpingectomy Group Error bars denote 95% CIs.

Figure 3 shows projections of serous cancers in the OS group if they arise at the same rate as those in the control group. Because there were no serous cancers in the OS group, we cannot estimate exactly how many of these serous cancers will be prevented. If OS were not performed, we would expect an estimated 36.9 (95% CI, 12.2-127.7) serous cancers by 2022 and 45.1 (95% CI, 14.7-157.5) cancers by 2027. These numbers do not account for additional individuals being added to the OS group.

## Discussion

The realization that the fallopian tube fimbriae is the tissue of origin for most HGSCs opened the door for OS as a primary ovarian cancer prevention strategy.^8^ Prevention of ovarian cancer seems more critical today than ever as the largest screening trial found that although a stage shift was achieved, there was no mortality benefit.^24^ In BC in 2010, a population-wide primary prevention campaign was initiated to remove the fallopian tubes of individuals at general population risk for ovarian cancer when they were undergoing hysterectomy for benign indications or seeking tubal ligation. The acceptability, safety, and cost-effectiveness of this OS campaign has already been established.^13,14,15,16^ In this cohort study, we now present data strongly suggesting that OS is effective as an ovarian cancer primary prevention strategy at the population level. There was not a single serous ovarian cancer in the OS group, which was significantly fewer than the slightly more than 5 that were expected. We have further shown that the OS group had the same risk of breast and colorectal cancers compared with the control group, indicating that the lack of ovarian cancers in the OS groups is unlikely to be associated with selection bias. The rates of common risk and protective factors for the OS group place them at slightly higher risk of ovarian cancer (eg, lower parity, lower gravidity, and higher age), indicating that our results are unlikely to be explained by confounding.

There were 15 serous cancers observed in the control group, and our calculations show that as we continue follow-up, there will be 45.1 serous ovarian cancers in this group by 2027. It is difficult to determine the preventable fraction given that we did not observe any serous cancers in our OS group. The least conservative interpretation would be that OS prevents all serous cancers, but more realistically it is probably more in line with the prevention achieved by risk-reducing salpingo-oophorectomy in patients with a *BRCA* variant, which is on the order of 80%.^5^ For example, if an estimated 200 000 individuals underwent hysterectomy without salpingectomy and a tubal ligation (instead of an OS) in Canada between 2011 and 2016, then on the basis of a 1% lifetime risk of HGSC and an assumed 80% effectiveness of OS, 1600 future cases of HGSC could theoretically have been prevented. Thus, although the numbers presented here are small, uptake of OS on a larger scale could be associated with the incidence of HGSCs nationally and internationally.

These findings may also further our understanding of the origin for HGSCs. Twenty years ago in a provocative editorial,^25^ it was suggested that the ovarian surface epithelium may not be the tissue of origin for ovarian carcinomas. Shortly afterward, detailed analysis of fallopian tubes and ovaries from *BRCA* variant carriers found tubal dysplastic lesions, now called serous tubal intraepithelial carcinomas, but no ovarian pathology.^26^ Systematic analysis of fallopian tubes and ovaries in many studies has since shown that serous tubal intraepithelial carcinomas are found in individuals with *BRCA* variants, also alongside sporadic and incidental HGSCs but not in the general population.^7,27,28,29,30^ Genomic studies have confirmed the clonal association between serous tubal intraepithelial carcinomas and HGSC, and transgenic mice with mutated fallopian tube cells produce histologically perfect HGSCs.^31,32^ However, there are data supporting potential primary ovarian origins of some HGSCs, including mouse models and expression data; thus, it is possible that some HGSCs arise from ovarian surface epithelium or endosalpingiosis.^33,34^ However, despite detailed analysis in many studies, credible ovarian HGSC precursors have not been described. Ultimately, expansion of the data presented here will determine the relative portion of HGSCs that originate in the fallopian tube vs the ovary.

Our findings are consistent with previous epidemiological research from the US, Denmark, and Sweden,^17,18,19^ which studied the association between excisional tubal surgery or salpingectomy and the risk of ovarian cancer among individuals with a medical indication for these procedures. The observed relative risks showed a 42% to 65% reduction in risk of ovarian cancer for individuals who underwent a major surgical procedure involving the fallopian tube.^17,18,19^ Furthermore, tubal ligation has long been recognized as being inversely associated with ovarian cancer risk, although the magnitude of the association is lower than those for the more extensive fallopian tube procedures mentioned already.^35^

### Limitations

Our work has some important limitations, including that these are observational data and not derived from a randomized clinical trial; thus, selection factors could introduce bias. However, the Table illustrates that there are few differences between the OS group and control group with respect to the most well-known risk and protective factors for ovarian cancer (eg, parity, oral contraceptive pill use, *BRCA* variant status, and endometriosis), and the differences that do exist would bias toward increased risk for ovarian cancer in the OS group. Although it remains possible that there are important unmeasured differences between the groups, such as lifestyle factors that are associated with cancer, the findings of no difference between the observed and expected numbers of breast and colorectal cancers in the OS group suggest that selection bias is unlikely to explain these results. The study was limited by the small number of cancers and relatively short follow-up time in our groups. Although uptake of OS has been substantial in BC, the province has a relatively small population (approximately 5 million), and the surgical procedures at which OS is performed occur at young mean ages. Thus, our numbers of ovarian cancers were small, making it impossible to run Cox proportional hazards models controlling for potential confounders. In addition, although the preliminary data suggest that there are no indicators of an earlier age of onset of menopause, it is time to conduct a long-term follow-up study on the age of onset of menopause as self-reported by those who undergo OS or a control surgery. Given the reduction in observed ovarian cancer compared with expected, which strongly supports reduced risk of ovarian cancer following OS, it is important to ensure OS does not alter the age of onset of menopause.

## Conclusions

This study found significantly smaller numbers of observed ovarian cancers compared with expected numbers for patients who underwent prophylactic OS at the time of hysterectomy or instead of tubal ligation. Most professional gynecological societies around the world have recommended consideration of OS. Our findings strengthen the evidence for presenting this option to patients at average risk of ovarian cancer. These data may also aid in patient decision-making around contraception options following childbearing.

## Notes

Supplement. **eTable.** Additional Details From Registry Sources
eReferences
