# Supplementary material for: Outcomes From Opportunistic Salpingectomy for Ovarian Cancer Prevention
Source: JAMA Netw Open. 2022 Feb 9;5(2):e2147343. doi: 10.1001/jamanetworkopen.2021.47343 (PMC8829665; doi:10.1001/jamanetworkopen.2021.47343)
Supplement: Supplement. — eTable. Additional Details From Registry Sources eReferences [file jamanetwopen-e2147343-s001.pdf]

Supplemental Online Content

Hanley GE, Pearce CL, Talhouk A, et al. Outcomes from opportunistic salpingectomy for ovarian cancer prevention. *JAMA Netw Open*. 2022;5(2):e2147343.
doi:10.1001/jamanetworkopen.2021.47343

eTable. Additional Details From Registry Sources
eReferences

This supplemental material has been provided by the authors to give readers additional information about their work.

© 2022 Hanley GE et al. *JAMA Network Open.*

eTable: Additional Details From Registry Sources

Dataset	Data contained in dataset	Years
Consolidation File[1]	BC health insurance registration information for the entire population of the province	2008-2017
Discharge Abstract Database[2]	All hospital stays and day surgeries performed in the province<br>• Surgical procedures undertaken<br>• ICD-10 diagnostic codes	2008-2017
BC Cancer Registry[3]	All cancer diagnosed in the province<br>• ICD for Oncology Diagnostic codes<br>• ICD-O morphology codes<br>• Date of diagnosis	2008-2017
BC Cancer Agency Screening Program data[4]	All individuals tested for a BRCA mutation in the province<br>• Test result<br>• Test date<br>• Result disclosure date	1994-2014

eReferences:

1. British Columbia Ministry of Health (2016): Consolidation File (MSP Registration & Premium Billing). V2. Population Data BC. Data Extract. MOH (2016). http://www.popdata.bc.ca/data/consolidation. Accessed August 11, 2021.
2. Canadian institute for health information. 2017. Discharge abstract database (hospital separations). V2. population data BC. data extract. MOH (2017). https://www.popdata.bc.ca/data/health/dad.
3. BC cancer agency registry data (2017). V2. Population data BC [publisher]. Data extract. BC cancer. https://www.popdata.bc.ca/data/health/bccancer
4. BC cancer agency screening program data (2017). Population data BC [publisher]. Data extract. BC cancer. https://www.popdata.bc.ca/data/health/bccancer

© 2022 Hanley GE et al. *JAMA Network Open.*
